# Impact of Tigecycline’s MIC in the Outcome of Critically Ill Patients with Carbapenemase-Producing *Klebsiella pneumoniae* Bacteraemia Treated with Tigecycline Monotherapy—Validation of 2019′s EUCAST Proposed Breakpoint Changes

**DOI:** 10.3390/antibiotics9110828

**Published:** 2020-11-19

**Authors:** Matthaios Papadimitriou-Olivgeris, Christina Bartzavali, Alexandra Nikolopoulou, Fevronia Kolonitsiou, Virginia Mplani, Iris Spiliopoulou, Myrto Christofidou, Fotini Fligou, Markos Marangos

**Affiliations:** 1 Division of Infectious Diseases, School of Medicine, University of Patras, 265 04 Patras, Greece; marangos@upatras.gr; 2 Department of Microbiology, School of Medicine, University of Patras, 265 04 Patras, Greece; chrisbartzavali@gmail.com (C.B.); kolonits@upatras.gr (F.K.); spiliopl@upatras.gr (I.S.); christof@upatras.gr (M.C.); 3 Anesthesiology and Critical Care Medicine, School of Medicine, University of Patras, 265 04 Patras, Greece; alex.nikolo.1981@gmail.com (A.N.); Virginiamplani@yahoo.gr (V.M.); fflig@yahoo.com (F.F.)

**Keywords:** tigecycline, bloodstream infection, carbapenemase, carbapenem-resistance, ceftazidime/avibactam, colistin, mortality

## Abstract

Background: Tigecycline is a therapeutic option for carbapenemase-producing *Klebsiella pneumoniae* (CP-Kp). Our aim was to evaluate the impact of the tigecycline’s minimum inhibitory concentration (MIC) in the outcome of patients with CP-Kp bacteraemia treated with tigecycline monotherapy. Methods: Patients with monomicrobial bacteraemia due to CP-Kp that received appropriate targeted monotherapy or no appropriate treatment were included. Primary outcome was 30-day mortality. MICs of meropenem, tigecycline, and ceftazidime/avibactam were determined by Etest, whereas for colistin, the broth microdilution method was applied. PCR for *bla*_KPC_, *bla*_VIM_, *bla*_NDM_, and *bla*_OXA_ genes was applied. Results: Among 302 CP-Kp bacteraemias, 32 isolates (10.6%) showed MICs of tigecycline ≤ 0.5 mg/L, whereas 177 (58.6%) showed MICs that were 0.75–2 mg/L. Colistin and aminoglycoside susceptibility was observed in 43.0% and 23.8% of isolates, respectively. The majority of isolates carried *bla*_KPC_ (249; 82.5%), followed by *bla*_VIM_ (26; 8.6%), both *bla*_KPC_ and *bla*_VIM_ (16; 5.3%), and *bla*_NDM_ (11; 3.6%). Fifteen patients with tigecycline MIC ≤ 0.5 mg/L and 55 with MIC 0.75–2 mg/L were treated with tigecycline monotherapy; 30-day mortality was 20.0% and 50.9%, respectively (*p* = 0.042). Mortality of 150 patients that received other antimicrobials was 24.7%; among 82 patients that received no appropriate treatment, mortality was 39.0%. No difference in 30-day mortality was observed between patients that received tigecycline (MIC ≤ 0.5 mg/L) or other antimicrobials. Conclusion: Tigecycline monotherapy was as efficacious as other antimicrobials in the treatment of bloodstream infections due to CP-Kp isolates with a tigecycline’s MIC ≤ 0.5 mg/L.

## 1. Introduction

Carbapenemase-producing *Klebsiella pneumoniae* (CP-Kp) has become a significant global public health challenge [1]. The arrival of novel beta-lactam/beta-lactamase inhibitor combinations, such as ceftazidime/avibactam, meropenem/vaborbactam, imipenem/cilastatin/relebactam, the siderophore cephalosporin, cefiderocol, and a next-generation aminoglycoside, plazomicin, has increased the available treating options in our arsenal, improving the outcome of such infections [2]. Previously, the available antimicrobial treatment options were colistin, tigecycline, aminoglycosides, fosfomycin, and carbapenems [3,4]. Combination therapy has been proposed as the best choice, but there are no clear data showing which combination therapy is superior [5].

Tigecycline is the first member of the glycylcycline class, has a broad spectrum of antibacterial activity, and achieves adequate levels into different tissues [6]. It has been approved for community-acquired pneumonia, skin and soft-tissue, and intraabdominal infections [6]. The use of tigecycline in bacteremia is controversial because of its low serum levels with standard dosing [7]. Despite such limitation, tigecycline is a useful alternative for the treatment of infections due to CP-Kp and has been shown to be an effective and safe drug for the treatment of severe CP-Kp infections [8,9]. In observational studies, tigecycline was equally effective to other options (colistin, aminoglycosides, carbapenems) even when used as monotherapy [8,9].

In 2019, the European Committee on Antimicrobial Susceptibility Testing (EUCAST) revised the minimum inhibitory concentration (MIC) breakpoints for tigecycline [10]; prior to 2019, an isolate was considered susceptible if the MIC was ≤ 1 mg/L and resistant if MIC was > 2 mg/L [11]; since 2019, an isolate with MIC > 0.5 mg/L has been considered resistant [10]. This revision rendered most of the isolates that were previously considered as susceptible to be considered resistant [12].

The aim of the present study was to evaluate the impact of the tigecycline’s MIC in the outcome of critically ill patients with CP-Kp bacteraemia treated with tigecycline monotherapy.

## 2. Results

In total, 302 episodes of monomicrobial bloodstream infections (BSIs) due to CP-Kp were included. Most BSIs were primary (131; 43.4%) and catheter-related (111; 36.8%); the remaining bacteraemias were associated with ventilator-associated pneumonia (24; 7.9%), abdominal infection (22; 7.3%), meningitis (7; 2.3%), urinary tract infection (6; 2.0%) and deep surgical site infection (1; 0.3%). The majority carried bla_KPC_ (249; 82.5%), followed by bla_VIM_ (26; 8.6%), both bla_KPC_ and bla_VIM_ (16; 5.3%), and bla_NDM_ (11; 3.6%) (Supplementary Materials).

Two isolates (0.7%) had MIC ≤ 8 mg/L to tested carbapenem (imipenem, meropenem). Concerning tigecycline, 32 isolates (10.6%) showed MIC ≤ 0.5 mg/L and 177 (58.6%) MICs ranging from 0.75 to 2 mg/L (Table 1). Susceptibility rates for aztreonam, sulfamethoxazole-trimethoprim, and ciprofloxacin were 2.3%, 4.3%, and 0.7%, respectively. Colistin and aminoglycoside susceptibility was observed in 43.0% and 23.8% of isolates, respectively. Fosfomycin and ceftazidime/avibactam were tested in 58 and 24 isolates, respectively; among them, 29 (50.0%) and 21 (87.5%) were susceptible.

Group A (tigecycline monotherapy; MIC of tigecycline ≤ 0.5 mg/L) and B (tigecycline monotherapy; MIC of tigecycline 0.75–2 mg/L) included 15 and 55 patients, respectively; the 30-day mortality was 20.0% and 50.9%, respectively. Group C (appropriate targeted monotherapy other than tigecycline) included 150 patients and had a 30-day mortality of 24.7%; the repartition of antimicrobials received was 111 colistin, 29 aminoglycoside, 8 ceftazidime/avibactam, and 2 carbapenems (MIC of imipenem and meropenem ≤ 4 mg/L). Group D (no appropriate targeted therapy) included 82 patients and had a 30-day mortality of 39.0%. The univariate analyses comparing different groups are shown in Table 2. Compared with Group A, Group B showed a statistically higher mortality (*p* = 0.042), while no difference in types of infections or septic shock occurrence or comorbidities was observed. No difference in 30-day mortality was observed among patients in Groups A and C. In addition, patients receiving appropriate monotherapy (Group A and C) had significantly lower 30-day mortality (*p* < 0.001) as compared to those that did not (Group B and D).

Univariate and multivariate analyses among Group A and Group B patients of predictors of 30-day mortality are shown in Table 3. Multivariate analysis revealed septic shock (*p* = 0.001; Odds Ratio 7.834, 95% Confidence Interval 2.343–26.198) as the sole independent predictors of mortality.

## 3. Discussion

In the last decades, there has been an important paucity of agents for adequately treating patients with CP-Kp bacteraemia [4]. Before the revision of tigecycline’s breakpoints, resistance rates to tigecycline were lower than other treatment options, such as colistin or aminoglycosides, leading to its wide use [3,12,13]. In 2019, the revision of tigecycline’s breakpoints resulted in a significant change of its susceptibility rates; in the present study, 89.4% of isolates were resistant according to 2019’s EUCAST breakpoints, whereas if the previous breakpoints were used, the resistance rate would drop to 30.8%. The latter rate represented the resistance rate reported in other studies conducted before the change of breakpoints [13,14].

The benefit of combination treatment over monotherapy remains a matter of debate when treating such infections, with some studies favoring the use of combination treatment, especially in critically ill patients [9,13,14]. Concerning monotherapy, tigecycline was considered as efficacious as other options for the treatment of CP-Kp infections [8,9,15]. Moreover, in a meta-analysis, in the subgroup of 398 KPC-producing *K. pneumoniae* bacteraemias, tigecycline was better than the other options [9].

Our data validate the change proposed by EUCAST in 2019, since the outcome of bacteraemias treated with tigecycline with MICs between 0.75 and 2 mg/L was worse than that of with MICs ≤ 0.5 mg/L, and comparable to those receiving no appropriate targeted treatment [10]. If the isolate’s MIC was ≤0.5 mg/L, tigecycline monotherapy was as efficacious as monotherapy with other treatment options (colistin, aminoglycoside, ceftazidime/avibactam, or carbapenems).

A main concern regarding the use of tigecycline to treat CP-Kp bacteraemias is the suboptimal concentrations, which could be overcome by increasing the dose, leading to better outcomes. [7] While this can be true for some types of infections, such as intra-abdominal or lower respiratory tract infections, bacteraemias represent difficult-to-treat infections, especially in critically ill patients. In an in vitro model, the standard tigecycline dose (100 mg/day) could be sufficient to treat bacteraemias by isolates with MICs < 0.06 mg/L, while a double dose (200 mg/day) was necessary for isolates with MICs of 0.125 to 0.25 mg/L [16]. Thus, the doses administered would not be sufficient to treat the majority of patients with CP-Kp bacteraemia [3,13,14].

This study has several limitations. It is a retrospective study in a Greek ICU with a moderate number of patients. The number of patients that received tigecycline for bacteraemia due to a CP-Kp isolate with tigecycline’s MIC ≤ 0.5 mg/L was small. While tigecycline was compared to other antimicrobials combined, no analysis was performed separately, since most of the patients in Group C received colistin and the other options (aminoglycoside, ceftazidime/avibactam, carbapenems) were underrepresented.

## 4. Materials and Methods

This retrospective study was carried out in the intensive care unit (ICU) of the University General Hospital of Patras (UGHP), Greece, during a ten-year period (2010–2019). The Ethical Committee of the UGHP approved the study (No 858).

Patients with a monomicrobial bacteraemia due to CP-Kp that received appropriate targeted monotherapy or no appropriate targeted treatment were included in the study. Those who received two or more appropriate antimicrobials were excluded. Groups A and B comprised of patients treated with tigecycline monotherapy, of which the infecting isolate had MICs of tigecycline ≤ 0.5 mg/L and 0.75–2 mg/L, respectively. Group C included patients treated with appropriate targeted monotherapy of colistin, aminoglycoside, ceftazidime/avibactam, or carbapenem, while Group D included patients that did not receive appropriate targeted therapy. Multiple episodes of bacteraemia from the same patient were included if a duration of at least two months occurred between two episodes.

Primary outcome was 30-day mortality. Data (epidemiological, comorbidities, antimicrobial administration, types of infection, and outcome) were obtained from patients’ chart reviews and the ICU computerized database (Criticus^TM^, University of Patras, Patras, Greece). Primary or secondary BSI was determined in accordance to the Centers for Disease Control and Prevention definition [17]. Infection was categorized as sepsis or septic shock according to new sepsis definition [18]. The date of collection of the first positive blood culture was defined as infection onset.

*K. pneumoniae* isolates from clinical specimens of patients hospitalized in UGHP were identified by the Vitek 2 Advanced Expert System (bioMérieux, Marcy-l’Étoile, France). Antimicrobial susceptibility testing was performed by the agar disk diffusion method against imipenem, meropenem, aztreonam, amikacin, gentamicin, sulfamethoxazole-trimethoprim, and ciprofloxacin. Minimum inhibitory concentrations (MICs) of imipenem, meropenem, tigecycline, fosfomycin, and ceftazidime/avibactam were determined by Etest (bioMérieux, Marcy-l’Étoile, France), whereas the MIC of colistin was determined by the broth microdilution method according to EUCAST methodology. EUCAST criteria were applied to interpret susceptibility result [10]. *bla*_VIM_, *bla*_IMP_, *bla*_KPC_, *bla*_NDM_, and *bla*_OXA_ were detected by PCR [19,20].

Data analysis was performed with SPSS version 23.0 (SPSS, Chicago, IL, USA). Fisher exact test or the *chi*^2^ test was used for categorical variables and Mann–Whitney *U*-test for continuous ones. Multiple logistic regression analysis was used to identify independent predictors of 30-day mortality. A *p* value < 0.05 was considered significant.

## 5. Conclusions

Tigecycline was as efficacious as other antimicrobials for the treatment of bacteraemia due to CP-Kp isolate with an MIC for tigecycline ≤ 0.5 mg/L. When tigecycline’s MIC ranged from 0.75 to 2 mg/L, patients’ clinical outcome was comparable to patients that received no appropriate antimicrobial treatment, thus affirming the proposed changes from EUCAST. Tigecycline can be used as monotherapy only if tigecycline’s MIC is ≤0.5 mg/L.

## Figures and Tables

**Table 1:** Minimum inhibitory concentration (MIC) distribution and susceptibility of 302 carbapenemase-producing *Klebsiella pneumoniae* isolates to different antimicrobials according to European Committee on Antimicrobial Susceptibility Testing (EUCAST) guidelines.

	MIC (mg/L)														EUCAST	
	0.125	0.25	0.38	0.5	0.75	1	1.5	2	3	4	6	8	12	32	S (%)	R (%)
Tigecycline	1	3	6	22	**20**	**52**	**52**	**53**	**18**	**47**	**6**	**12**	**3**	**7**	32 (10.6)	270 (89.4)

Resistant isolates according to EUCAST appear in bold. S: susceptible; R: resistant.

**Table 2:** Univariate analyses of characteristics of patients depending on received antibiotic treatment among patients with carbapenemase-producing *K. pneumoniae* (CP-Kp) bloodstream infection (BSI) during intensive care unit (ICU) hospitalization.

Characteristics	Group A (*N* = 15) Tigecycline Monotherapy (MIC ≤ 0.5 Mg/L)	Group B (*N* = 55) Tigecycline Monotherapy (MIC 0.75–2 Mg/L)	*p* ^a^	Group C (*N* = 150) ^b^ Monotherapy Other Than Tigecycline	*p* ^c^	Group D (*N* = 82) No Appropriate Treatment	*p* ^d^
Age (years)	47.3 ± 18.2	58.4 ± 17.9	0.063	55.1 ± 17.5	0.110	55.7 ± 17.1	0.310
Male gender	10 (66.7%)	38 (69.1%)	1.000	110 (73.3%)	0.556	57 (69.5%)	0.526
Charlson Comorbidity Index	2.3 ± 3.1	3.6 ± 3.7	0.095	3.5 ± 3.5	0.132	2.9 ± 3.2	0.734
Obesity	3 (20.0%)	18 (32.7%)	0.527	40 (26.7%)	0.716	25 (30.5%)	0.370
Infection data							
Days at risk	39.0 ± 69.1	26.5 ± 25.9	0.726	19.6 ± 27.0	0.134	28.5 ± 36.9	0.027
Type of bacteraemia							
Primary	6 (40.0%)	28 (50.9%)	0.564 ^e^	60 (40.0%)	1.000 ^e^	37 (45.1%)	0.202 ^e^
Catheter-related	4 (26.7%)	15 (27.3%)		65 (43.3%)		27 (32.9%)	
Other ^f^	5 (33.3%)	12 (21.8%)		25 (16.7%)		18 (22.0%)	
Septic shock	7 (46.7%)	34 (61.8%)	0.378	62 (41.3%)	0.786	32 (39.0%)	0.296
SAPS II upon onset of infection	39.5 ± 11.3	41.4 ± 13.1	0.784	39.9 ± 11.4	0.849	41.3 ± 13.2	0.626
SOFA score upon onset of infection	7.3 ± 4.0	8.4 ± 3.6	0.192	7.2 ± 3.3	0.823	7.4 ± 3.5	0.149
Hemofiltration	1 (6.7%)	4 (7.3%)	1.000	11 (7.3%)	1.000	8 (9.8%)	0.673
Outcome							
30-day mortality	3 (20.0%)	28 (50.9%)	0.042	37 (24.7%)	0.767	32 (39.0%)	<0.001

Data are number (%) of patients or mean ± standard deviation. SAPS II: Simplified Acute Physiology Score II; SOFA: Sequential Organ Failure Assessment. ^a^ Comparison between Group A and B. ^b^ 111 colistin, 29 aminoglycoside, 8 ceftazidime/avibactam, and 2 carbapenems. ^c^ Comparison between Group A and C. ^d^ Comparison between Groups A and C against Groups B and D. ^e^ Comparison of primary BSIs against secondary ones. ^f^ 24 ventilator-associated pneumonias, 22 abdominal infections, 7 meningitis, 6 urinary tract infections, and 1 deep surgical site infection.

**Table 3:** Univariate and multivariate analyses of predictors of 30-day mortality among Groups A and B patients with carbapenemase-producing *K. pneumoniae* (CP-Kp) bloodstream infection (BSI) during intensive care unit (ICU) hospitalization.

	Univariate Analysis			Multivariate Analysis	
Characteristics	Survivors (*N* = 39)	Non-Survivors (*N* = 31)	*p*	*p*	OR (95% CI)
Age (years)	53.4 ± 17.5	61.6 ± 16.3	0.001		
Male gender	27 (69.2%)	21 (67.7%)	1.000		
Charlson Comorbidity Index	2.9 ± 3.4	4.5 ± 3.4	0.016	0.227	1.100 (0.942–1.283)
Obesity	10 (25.6%)	11 (35.5%)	0.436		
Infection data					
Days at risk	25.0 ± 35.4	22.1 ± 25.5	0.636		
Type of bacteraemia					
Primary	20 (51.3%)	14 (45.2%)			
Catheter-related	14 (35.9%)	5 (16.1%)	0.104 ^a^		
Other ^b^	5 (13.7%)	12 (38.2%)			
Septic shock	15 (38.5%)	26 (83.9%)	<0.001	0.001	7.834 (2.343–26.198)
SAPS II upon onset of infection	37.4 ± 9.9	51.4 ± 13.0	0.001		
SOFA score upon onset of infection	6.4 ± 2.8	10.7 ± 3.4	<0.001		
Hemofiltration	2 (5.1%)	3 (9.7%)	1.000		
Tigecycline MIC ≤ 0.5 mg/L	12 (30.8%)	3 (9.7%)	0.042	0.069	0.242 (0.052–1.118)

Data are number (%) of patients or mean ± standard deviation. OR: odds ratio; CI: confidence interval; SAPS II: Simplified Acute Physiology Score II; SOFA: Sequential Organ Failure Assessment. ^a^ Comparison between catheter-related bacteraemia and all other types of infection. ^b^ Seven ventilator-associated pneumonias, five abdominal infections, three meningitis, one urinary tract infection, and one deep surgical site infection.

## Supplementary Materials

The following are available online at https://www.mdpi.com/2079-6382/9/11/828/s1.

## References

[1] Albiger B., Glasner C., Struelens M.J., Grundmann H., Monnet D.L. (2015). European survey of carbapenemase-Producing enterobacteriaceae working, g. carbapenemase-producing enterobacteriaceae in Europe: Assessment by national experts from 38 countries, May 2015. Eurosurveillance.

[2] Bassetti M., Vena A., Sepulcri C., Giacobbe D.R., Peghin M. (2020). Treatment of bloodstream infections due to gram-negative bacteria with difficult-to-treat resistance. Antibiotics.

[3] Papadimitriou-Olivgeris M., Fligou F., Bartzavali C., Zotou A., Spyropoulou A., Koutsileou K., Vamvakopoulou S., Sioulas N., Karamouzos V., Anastassiou E.D. (2017). Carbapenemase-producing *Klebsiella pneumoniae* bloodstream infection in critically ill patients: Risk factors and predictors of mortality. Eur. J. Clin. Microbiol. Infect. Dis..

[4] Karaiskos I., Lagou S., Pontikis K., Rapti V., Poulakou G. (2019). The “Old” and the “New” Antibiotics for MDR Gram-Negative Pathogens: For Whom, When, and How. Front. Public Health.

[5] Gutierrez-Gutierrez B., Salamanca E., de Cueto M., Hsueh P.R., Viale P., Pano-Pardo J.R., Venditti M., Tumbarello M., Daikos G., Canton R. (2017). Effect of appropriate combination therapy on mortality of patients with bloodstream infections due to carbapenemase-producing Enterobacteriaceae (INCREMENT): A retrospective cohort study. Lancet Infect. Dis..

[6] Stein G.E., Babinchak T. (2013). Tigecycline: An update. Diagn. Microbiol. Infect. Dis..

[7] Falagas M.E., Vardakas K.Z., Tsiveriotis K.P., Triarides N.A., Tansarli G.S. (2014). Effectiveness and safety of high-dose tigecycline-containing regimens for the treatment of severe bacterial infections. Int. J. Antimicrob. Agents.

[8] Ni W., Han Y., Liu J., Wei C., Zhao J., Cui J., Wang R., Liu Y. (2016). Tigecycline treatment for carbapenem-resistant enterobacteriaceae infections: A systematic review and meta-analysis. Medicine.

[9] Wang J., Pan Y., Shen J., Xu Y. (2017). The efficacy and safety of tigecycline for the treatment of bloodstream infections: A systematic review and meta-analysis. Ann. Clin. Microbiol. Antimicrob..

[10] The European Committee on Antimicrobial Susceptibility Testing (2019). Breakpoint Tables for Interpretation of MICs and Zone Diameters. Version 9.0. http://www.eucast.org.

[11] The European Committee on Antimicrobial Susceptibility Testing (2018). Breakpoint Tables for Interpretation of MICs and Zone Diameters. Version 8.1. http://www.eucast.org.

[12] Papadimitriou-Olivgeris M., Bartzavali C., Spyropoulou A., Lambropoulou A., Sioulas N., Vamvakopoulou S., Karpetas G., Spiliopoulou I., Vrettos T., Anastassiou E.D. (2018). Molecular epidemiology and risk factors for colistin- or tigecycline-resistant carbapenemase-producing *Klebsiella pneumoniae* bloodstream infection in critically ill patients during a 7-year period. Diagn. Microbiol. Infect. Dis..

[13] Kontopidou F., Giamarellou H., Katerelos P., Maragos A., Kioumis I., Trikka-Graphakos E., Valakis C., Maltezou H.C., on behalf of the Group for the Study of KPC-producing *Klebsiella pneumoniae* infections in intensive care units (2014). Infections caused by carbapenem-resistant *Klebsiella pneumoniae* among patients in intensive care units in Greece: A multi-centre study on clinical outcome and therapeutic options. Clin. Microbiol. Infect..

[14] Tumbarello M., Trecarichi E.M., De Rosa F.G., Giannella M., Giacobbe D.R., Bassetti M., Losito A.R., Bartoletti M., Del Bono V., Corcione S. (2015). Infections caused by KPC-producing *Klebsiella pneumoniae*: Differences in therapy and mortality in a multicentre study. J. Antimicrob. Chemother..

[15] Satlin M.J., Kubin C.J., Blumenthal J.S., Cohen A.B., Furuya E.Y., Wilson S.J., Jenkins S.G., Calfee D.P. (2011). Comparative effectiveness of aminoglycosides, polymyxin B, and tigecycline for clearance of carbapenem-resistant *Klebsiella pneumoniae* from urine. Antimicrob. Agents Chemother..

[16] Tsala M., Vourli S., Daikos G.L., Tsakris A., Zerva L., Mouton J.W., Meletiadis J. (2017). Impact of bacterial load on pharmacodynamics and susceptibility breakpoints for tigecycline and *Klebsiella pneumoniae*. J. Antimicrob. Chemother..

[17] Horan T.C., Andrus M., Dudeck M.A. (2008). CDC/NHSN surveillance definition of health care-associated infection and criteria for specific types of infections in the acute care setting. Am. J. Infect. Control.

[18] Singer M., Deutschman C.S., Seymour C.W., Shankar-Hari M., Annane D., Bauer M., Bellomo R., Bernard G.R., Chiche J.D., Coopersmith C.M. (2016). The third international consensus definitions for Sepsis and Septic Shock (Sepsis-3). JAMA.

[19] Queenan A.M., Bush K. (2007). Carbapenemases: The versatile beta-lactamases. Clin. Microbiol. Rev..

[20] Nordmann P., Poirel L., Carrer A., Toleman M.A., Walsh T.R. (2011). How to detect NDM-1 producers. J. Clin. Microbiol..

